# Evaluation of Methods to Improve the Extraction and Recovery of DNA from Cotton Swabs for Forensic Analysis

**DOI:** 10.1371/journal.pone.0116351

**Published:** 2014-12-30

**Authors:** Michael S. Adamowicz, Dominique M. Stasulli, Emily M. Sobestanovich, Todd W. Bille

**Affiliations:** 1 Forensic Science Department, Henry C. Lee College of Criminal Justice & Forensic Sciences, University of New Haven, West Haven, Connecticut, United States of America; 2 National Laboratory Center, Bureau of Alcohol, Tobacco, Firearms and Explosives, Ammendale, Maryland, United States of America; U.S. Geological Survey, United States of America

## Abstract

Samples for forensic DNA analysis are often collected from a wide variety of objects using cotton or nylon tipped swabs. Testing has shown that significant quantities of DNA are retained on the swab, however, and subsequently lost. When processing evidentiary samples, the recovery of the maximum amount of available DNA is critical, potentially dictating whether a usable profile can be derived from a piece of evidence or not. The QIAamp DNA Investigator extraction kit was used with its recommended protocol for swabs (one hour incubation at 56°C) as a baseline. Results indicate that over 50% of the recoverable DNA may be retained on the cotton swab tip, or otherwise lost, for both blood and buccal cell samples when using this protocol. The protocol’s incubation time and temperature were altered, as was incubating while shaking or stationary to test for increases in recovery efficiency. An additional step was then tested that included periodic re-suspension of the swab tip in the extraction buffer during incubation. Aliquots of liquid blood or a buccal cell suspension were deposited and dried on cotton swabs and compared with swab-less controls. The concentration of DNA in each extract was quantified and STR analysis was performed to assess the quality of the extracted DNA. Stationary incubations and those performed at 65°C did not result in significant gains in DNA yield. Samples incubated for 24 hours yielded less DNA. Increased yields were observed with three and 18 hour incubation periods. Increases in DNA yields were also observed using a swab re-suspension method for both cell types. The swab re-suspension method yielded an average two-fold increase in recovered DNA yield with buccal cells and an average three-fold increase with blood cells. These findings demonstrate that more of the DNA collected on swabs can be recovered with specific protocol alterations.

## Introduction

The cotton swab has long been a basic and essential tool for collecting deoxyribonucleic acid (DNA) evidence for forensic casework analysis [1], [2]. However, a challenge to this analysis has been the quantity of usable sample recovered from evidentiary items for short tandem repeat (STR) analysis using the polymerase chain reaction (PCR). Many factors can affect the recovery of a DNA sample, including the type of sample such as body fluids and epithelial cells [3], the type of evidence being examined such as skin [4], [5], fingernails [6], [7], sexual assault kits [8], and improvised explosive devices [9], [10]. Studies have also indicated that the substrate that the sample is being collected from can have an effect on the quantity of human DNA recovered for analysis [11], [12]. The recovered quantity is a critical factor in the success of forensic DNA testing, as too little may result in stochastic amplification and the loss of allelic or locus signal due to insufficient template. This is termed low-level DNA testing and has been reviewed extensively [13].

There are multiple methods used with low-level evidentiary samples to increase the number of detected STR alleles or to increase allelic peak signals to levels that can be reliably analyzed. Validated protocols exist for concentrating samples [14], amplification with reduced volume [15], post-PCR purification [16], increased number of PCR cycles [17], and increased capillary injection settings [18]. While often effective in improving the DNA profile peak numbers and heights, these methods can lead to a variety of artifacts, including spectral disruptions, increased baseline and stutter values, and allele drop-in [18]. A more reliable method to improve DNA profile results is to simply start with more DNA through improved collection.

A variety of methodologies intended to increase the quantity and/or quality of evidentiary DNA have been used. Although many DNA samples are extracted directly from the solid support that they are found on, such as clothing items, upholstery, paper, chewing gum, and cigarette butts, direct extraction can carry over high concentrations of a variety of molecules which are inhibitory to the Taq DNA polymerase enzyme required for amplification via PCR from items such as bone, leather, and soil [19]. Opel et al. [19] examined the PCR inhibition mechanisms of humic acid, tannic acid, and indigo dye. All of these compounds can be found in evidentiary items submitted to forensic laboratories for DNA testing. In order to reduce the carry-over of inhibitory agents and preserve sample for further testing, biological material is often collected from the items using some type of intermediary device such as a swab that will retain the DNA until processing and analysis begin. While a variety of collection approaches have been used, such as self-adhesive security seals [20] and adhesive tape [21], a common process is still the use of cotton tipped swabs to gather and retain the samples for transport to the laboratory and storage of the material in a concentrated form [1], [2], [3].

The ubiquitous method for collecting biological evidence with a cotton tipped swab has been to wet the tip with water, usually sterile and deionized, and to rub or roll the tip on and over the area of interest. Sweet et al. described an improved method using a wet swabbing immediately followed by a dry swabbing that increased the yield of recovered DNA from saliva placed on human skin [22]. This technique was then shown to be more effective than using a single wet swab for collecting low quantities of DNA from touched evidence by Pang and Cheung [23]. The role of the wetting agent used with the swab has also been examined, with the addition of a detergent solution rather than pure water yielding higher quantities of recovered DNA [24].

Recently there have been several new types of swabs that have become available to forensic casework. The efficacy of DNA recovery using nylon flocked swabs was compared to that of traditional cotton swabs in post-coital vaginal samples [25] as well as with high-quality and decreased-quality samples [26]. While the nylon flocked swabs were reported as having better performance in the collection and release of sperm cell DNA, they were less efficient than cotton swabs for the vaginal epithelial cells and had slower adsorption rates and longer drying times than cotton swabs [25]. Brownlow et al. [26] also reported complications with the adsorption rate of the nylon flocked swabs, as well as variations in DNA yield between nylon flocked and cotton swabs depending on the extraction method used. Marshall et al. [27] compared the Copan 4N6FLOQSwab (Brescia, Italy) nylon flocked swab to the X-Swab (Diomics Corporation, La Jolla, CA). They found that the samples collected with the X-Swab had superior DNA yield and higher average peak heights to those collected with the Copan 4N6FLOQSwab [27].

While there has been much attention and effort applied to the type of swab used to collect DNA evidence from many types of surfaces and substrates combined with multiple extraction methods, there has been less work performed on modifying extraction methods to enhance the release of the DNA carried in the fibers of the swab. This study was designed to address this area by assessing multiple extraction variables such as incubation time, temperature, and washing the fibers to find if any gains in overall DNA yield could be achieved. The alterations to the standard extraction protocol were tested individually and then in combinations. Cotton tipped swabs were used as they are still the least expensive and one of the most common collection devices, however the protocols described could be performed with any type of durable swab and most of the extraction methods commonly used in forensic DNA laboratories. While not all of the protocol modifications increased DNA recovery, some showed substantial gains.

## Materials and Methods

Buccal swabs and blood samples were collected from a participant who volunteered to donate materials for this study. All samples were collected according to the University of New Haven Institutional Review Board’s (IRB) approved policies for ethical standards and methods for human testing. Sample collection and experimentation commenced only after the IRB review process was completed and express written approval for this study was received from the UNH IRB and with the participant’s written informed consent.

### Sample Collection and Storage

Sterile cotton tipped applicator swabs (Puritan Medical Products, Guilford, ME) were used throughout this study. Epithelial cell suspensions were created by collecting twenty buccal swabs, and combining them in groups of four. After vigorously rubbing the inner cheek and gum areas of the mouth, the cotton tips of the fresh swabs from each group were completely cut away from the wooden sticks and placed together in approximately 400 µL of 0.1 M Tris (Fisher Scientific, Fair Lawn, NJ) buffer (pH 7.5) in a 2 mL plastic flip-top tube and shaken for 10 minutes at 900 rpm to release the epithelial cells. The swab tips were then removed from the buffer and centrifuged in a DNA IQ Spin Basket (Promega, Madison, WI) to collect all of the available sample. The cell suspensions were then brought up to a volume of 500 µL each with 0.1 M Tris buffer (pH 7.5), checked microscopically to confirm the presence of intact epithelia. The cell suspensions were then further separated into 50 µL volumes and these single experiment aliquots were frozen at −20°C until used. A 20 µL volume from these aliquots consistently yielded 1–1.5 ng/µL of extracted DNA. Freshly collected whole blood was separated into single experimental aliquots of 50 µL and frozen until used. Samples for the aging experiments were created as follows; triplicate swabs were prepared for each time point and extraction condition, dried overnight, and stored for one week, one month (31 days), three months, or six months at 4°C.

### DNA Extractions

All of the swab samples tested were spotted with either a 20 µL aliquot of the liquid buccal cell suspension or 10 µL of liquid blood. The swabs were then air dried overnight, unless otherwise specified. Blank swabs were also extracted for all conditions to serve as reagent blank negative controls. Swab-less controls consisted of the same volume of sample placed directly into the lysis buffer. DNA from all of the samples was extracted using the QIAamp DNA Investigator kit (Qiagen, Hilden, Germany) and the protocol for the “Isolation of total DNA from surface and buccal swabs” as a starting point [28]. All samples followed the volume specifications for a cotton or Dacron swab and carrier RNA was not used for this application. The samples were always eluted in a final volume of 50 µL of QIAamp buffer ATE (Qiagen, Hilden, Germany). Samples requiring agitation were shaken on an Eppendorf Thermomixer R (Eppendorf, Hamburg, Germany).

### Alterations to the Extraction Protocol

The QIAamp DNA Investigator kit standard extraction protocol for swabs specifies incubation in buffer QIAamp ATL (Qiagen, Hilden, Germany) in the presence of proteinase K at 56°C with shaking for at least one hour [28]. The parameters this study focused on were the time and temperature of incubation and agitating the sample using a thermomixer or incubating them while stationary. These parameters are easily altered, common to different DNA extraction methods such as organic solvent and silica/magnetic bead kits, and can be rapidly validated in laboratories. An additional step was added to the standard extraction protocol for the swabs that underwent “re-suspension”. During incubation, these swabs were removed from the QIAamp extraction buffer ATL and placed in a Promega DNA IQ Spin Basket (Madison, WI) which was fitted back into the extraction tube containing the liquid extract from the sample. The swabs were centrifuged for one minute at maximum speed (13,200 rpm) in an Eppendorf model 5415D centrifuge, collecting all of the liquid. The cotton swab tip was then returned into the extraction buffer and the incubation continued. The re-suspension process was repeated every 20 minutes with clean spin baskets for the duration of the incubation (one or three hours, depending on the experimental conditions). During the re-suspension process, no eluent was lost or discarded, it was always collected back into the sample. The parameters tested and their combinations are shown in Table 1. All experiments were conducted in duplicate or triplicate (specific number listed in Tables 1–3) and were intended to demonstrate initial trends in recoverable average DNA yields.

**Table 1:** Summary of extraction protocol alterations.

Incubation Time (hours)	Method	Incubation Temperature
1, 3, 18, and 24	Thermomixer at 900 rpm	56°C (N = 3 for 1 hour, N = 2 for 3, 18, and 24 hours)
1, 3, 18, and 24	Thermomixer at 900 rpm	65°C (N = 2)
1, 3, 18, and 24	Stationary	56°C (N = 2)
1, 3, 18, and 24	Stationary	65°C (N = 2)
1 and 3	Re-suspension + Thermomixer at 900 rpm	56°C (N = 3)
1 and 3	Re-suspension + Thermomixer at 900 rpm	65°C (N = 3)
1 and 3	Re-suspension + Stationary	56°C (N = 3)
1 and 3	Re-suspension + Stationary	65°C (N = 3)

**Table 2:** Average DNA quantitation values^1^ for experiments with buccal cells.

Incubation Conditions								
Incubation Time	Buccal Cells Shaken 56°C	Buccal Cells Stationary 56°C	Buccal Cells Shaken 65°C	Buccal Cells Stationary 65°C	Buccal Cells Shaken w/Re-suspension 56°C	Buccal Cells Stationary w/Re-suspension 56°C	Buccal Cells Shaken w/Re-suspension 65°C	Buccal Cells Stationary w/Re-suspension 65°C
1 Hour	0.54±0.06	0.26±0.02	0.20±0.03	0.17±0.04	1.32±0.51	0.37±0.08	0.50±0.09	0.32±0.02
3 Hours	0.31±0.07	0.30±0.02	0.60±0.01	0.13±0.02	0.71±0.25	0.87±0.15	0.85±0.06	0.75±0.10
18 Hours	0.44±0.11	0.38±0.07	0.65±0.01	0.43±0.06	-	-	-	-
24 Hours	0.29±0.04	0.11±0.02	0.15±0.04	0.17±0.09	-	-	-	-

1 All values are expressed in ng/µL. Extraction for one hour at 56°C with shaking without re-suspension N = 3. All other extractions without re-suspension N = 2. Extractions with re-suspension N = 3.

**Table 3:** Average DNA quantitation values^1^ for experiments with blood cells.

Incubation Conditions								
Incubation Time	Blood Cells Shaken 56°C	Blood Cells Stationary 56°C	Blood Cells Shaken 65°C	Blood Cells Stationary 65°C	Blood Cells Shaken w/Re-suspension 56°C	Blood Cells Stationary w/Re-suspension 56°C	Blood Cells Shaken w/Re-suspension 65°C	Blood Cells Stationary w/Re-suspension 65°C
1 Hour	0.64±0.33	0.56±0.05	0.49±0.10	0.89±0.10	1.87±0.42	1.56±0.13	1.20±0.30	1.30±0.18
3 Hours	1.45±0.33	0.56±0.17	1.47±0.02	1.09±0.03	2.10±0.27	2.6±0.35	2.11±0.19	2.18±0.05
18 Hours	1.18±0.15	0.65±0.14	0.78±0.18	1.06±0.33	-	-	-	-
24 Hours	0.32±0.10	0.24±0.01	0.08±0.03	0.07±0.02	-	-	-	-

1 All values are expressed in ng/µL. Extraction for one hour at 56°C with shaking without re-suspension N = 3. All other extractions without re-suspension N = 2. Extractions with re-suspension N = 3.

### Quantitation and Dilution

DNA extractions were quantified using an Applied Biosystems (Foster City, CA) 7500 real-time PCR system and the Applied Biosystems by Life Technologies (Foster City, CA) Quantifiler Human DNA Quantification Kit according to the manufacturer’s recommendations. Based on the quantitation results, any sample that yielded a concentration of DNA that was more than 1 ng/µL was diluted with sterile deionized water down to approximately 1 ng/µL prior to amplification.

### Amplification-AmpFlSTR Identifiler PCR Amplification Kit

All samples were amplified using the Applied Biosystems by Life Technologies (Foster City, CA) AmpFlSTR Identifiler kit following the manufacturer’s protocols [29]. The target quantity of DNA for amplification was 1–1.5 ng, however some samples yielded concentrations of DNA that fell below that value. In those cases the maximum volume of sample extract (10 µL) was used. The amplification reaction final volume was 25 µL and thermal cycling was performed in an Applied Biosystems GeneAmp PCR System 9700 using 9600 emulation mode for 28 cycles. All amplification sets included a positive control (cell line 9947A DNA, included in kit), a negative control (deionized water used for dilutions), and a reagent blank negative control (described previously).

### Capillary Electrophoresis

Sample amplified fragment separation and detection was performed on an Applied Biosystems 3730 Prism Genetic Analyzer. Samples were prepared for injection in a mixture of Applied Biosystems by Life Technologies HiDi formamide (8.7 µL/sample) and Applied Biosystems by Life Technologies GeneScan 500 LIZ size standard (0.3 µL/sample). A total volume of 10 µL (1 µL sample or allelic ladder and 9 µL formamide and size standard) was added to the appropriate wells for injection. All injections were performed at 3 kV for 5 seconds and sample amplicons were separated using Applied Biosystems by Life Technologies Performance Optimized Polymer (POP) 7. Injection results were analyzed using Applied Biosystems GeneMapper ID v3.2 software.

### Statistical Analysis

Experimental data were analyzed using GenStat 16^th^ ed. Software (VSN International Ltd). One- and two-way ANOVA tests were conducted to determine significant differences in the factors of the experiments. Values that reached significance are reported in the text and Supporting Information (SI) tables, those that did not can be found in SI tables only.

## Results

### Baseline Samples

**Figure 1:**
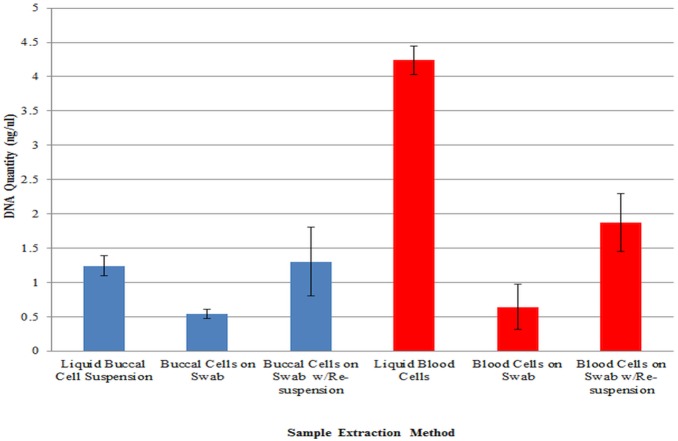
Comparison of average recovered DNA quantities from buccal cell suspension (20 µL) or blood (10 µL) dried onto cotton swabs versus liquid controls using the recommended protocol of incubation for 1 hour at 56°C with shaking (900 rpm). Results are also shown for samples dried onto cotton swabs and extracted after an incubation for 1 hour at 56°C with shaking (900 rpm) with swab re-suspension. N = 3.

In order to establish a baseline for the yield of DNA from cotton tipped swabs using the standard extraction protocol, a comparative experiment was performed measuring the quantity of DNA recovered from a set volume of blood or buccal cell suspension versus the same volume of each spotted and dried onto clean cotton swabs. Both blood and the buccal cell suspensions were used in all of the experiments to assess any differences that the two cell types may have in adsorption or release from the cotton fibers. Each cell type was run in triplicate for the baseline yield experiments. The results can be seen in Fig. 1. A 20 µL aliquot of the liquid buccal cell suspension had an average yield of 1.24±0.15 ng/µL DNA versus 0.54±0.06 ng/µL DNA for the same volume dried on a cotton swab, a difference which reached significance (F_(1,4)_ = 55.35, *p*-value = 0.002) (S1 Table). A volume of 10 µL of liquid blood was used due to an expectation of higher yield than the buccal cell suspension, which proved accurate. The blood sample yielded an average value of 4.24±0.21 ng/µL DNA while an equal volume extracted from a cotton swab resulted in 0.64±0.33 ng/µL DNA, a difference which also reached significance (F_(1,4)_ = 251.92, *p*-value<0.001) (S1 Table). These results indicated that considerable quantities of DNA were retained in the cotton fibers of the swabs, more than 50% of the buccal suspension DNA and over 80% of the blood DNA was not recovered with the standard extraction protocol.

### Shaken versus Stationary and Incubation Duration

Identical samples were incubated in QIAamp ATL buffer and proteinase K following the standard protocol (56°C, one hour) with the exception that one set was incubated with shaking at 900 rpm in a thermomixer and the other set was incubated stationary in a thermomixer. The average quantitation values for each set are shown in Table 2 (buccal cells) and Table 3 (blood cells).

The average yield of recovered DNA was increased with buccal cells when the samples were shaken at 900 rpm. The difference, however, was not significant (S2 Table) at an incubation time of one hour, with the average recovered DNA yields of 0.54±0.06 ng/µL for samples incubated with shaking and 0.26±0.02 ng/µL for samples incubated without shaking (Table 2). The experiment was repeated with increasing incubation times of three hours, 18 hours, and 24 hours. The average quantitation results for all of these conditions are shown in Table 2 and the only time point at which the average recovered DNA values, 0.29±0.04 ng/µL with shaking and 0.11±0.02 ng/µL without shaking, approached significance was at 24 hours (S2 Table). Shaken buccal cell samples showed a decrease in average DNA yield at the maximum incubation time, from 0.54±0.06 ng/µL at one hour to 0.29±0.04 ng/µL at 24 hours, which was significant (F_(1,4)_ = 19.55, *p*-value = 0.021) (S2 Table). The buccal cell samples that were incubated stationary showed an increase in average yield over time, up to 18 hours (0.38±0.07 ng/µL), and then a drop-off at 24 hours (0.11±0.02 ng/µL). The overall change in recovered DNA values for stationary samples, from one hour to 18 hours, did not reach significance, however the decrease in yield from 18 hours to 24 hours did (Table 2) (F_(1,3)_ = 121.07, *p*-value = 0.008) (S2 Table).

The average yield of recovered DNA was also increased with blood cells when the samples were shaken at 900 rpm (Table 3). The difference in yield, however, was again not significant between shaken and stationary at an incubation time of one hour (S3 Table). Although more DNA was recovered, on average, from samples that were shaken at both three and 18 hours of incubation than the same time points incubated without shaking (Table 3), neither comparison reached significance (S3 Table). Blood cells incubated with shaking showed an increased average yield at three hours (1.45±0.33 ng/µL) and 18 hours (1.18±0.15 ng/µL) over the average quantity recovered at one hour (0.64±0.33 ng/µL), while 24 hour incubations had the lowest average yield (0.32±0.10 ng/µL). Similar to the results with buccal cells, the average blood quantitation value changes from one hour up to 18 hours did not reach significance (S3 Table), however the average quantity yield with three hours of incubation (1.45±0.33 ng/µL) was increased when compared to that at 24 hours (0.32±0.10 ng/µL) (S3 Table). The recovered DNA quantitation values for the blood cell samples that were not shaken during incubation changed very little, with the highest average yield at 18 hours (0.65±0.14 ng/µL) and the lowest at 24 hours (0.24±0.01 ng/µL) (Table 3). The combined recovered yield differences from one hour to 24 hours of incubation while stationary did not reach significance (S3 Table).

### Incubation Temperature

Buccal and blood cell samples were again incubated in QIAamp ATL buffer and proteinase K with and without shaking for one, three, 18, or 24 hours, but the incubation temperature was changed from 56°C to 65°C. The resulting average quantitation values for each set are shown in Table 2 (buccal cells) and Table 3 (blood cells).

The results of increasing the incubation temperature with buccal cells were mixed. When the samples were incubated stationary at 65°C, average quantitation values were lower than those when stationary at 56°C for the one and three hour time points, but higher at the 18 and 24 hour time points (Table 2). When the samples were incubated with shaking at 900 rpm at 65°C, average quantitation values were lower than those at 56°C for the one and 24 hour time points and higher at three and 18 hours (Table 2). The combined buccal cell quantitation value differences between incubation conditions, shaking and stationary, at 56°C and 65°C were small, however, and did not show significance when time was not considered (S4 Table). However, the increase in buccal cell yields between the temperatures over time up to 18 hours of incubation with and without shaking did reach significance (F_Time(3,9)_ = 7.90, *p*-value = 0.007, F_Temp(1,9)_ = 0.06, *p*-value = 0.808, F_Int(3,9)_ = 12.98, *p*-value = 0.001) (S4 Table). Incubating the buccal cell samples longer than 18 hours usually showed decreased DNA yields. With the exception of the buccal cells incubated stationary at 65°C, 24 hour incubation periods yielded the lowest average quantitation values in each experimental buccal set (Table 2). The highest average yield of recovered DNA from buccal cells that were not re-suspended, was observed when the samples were incubated for 18 hours at 65°C with shaking at 900 rpm (0.65±0.01 ng/µL). This was a small increase over the standard protocol average value observed for samples incubated for one hour at 56°C with shaking at 900 rpm (0.54±0.06 ng/µL) (Table 2).

The experiments with blood cells incubated at 65°C also had mixed results. When the samples were incubated stationary at 65°C, average quantitation values were higher than those when stationary at 56°C for the one, three, and 18 hour time points, but lower at the 24 hours (Table 3). When the samples were incubated with shaking at 900 rpm at 65°C, average quantitation values were lower than those at 56°C for the one, 18, and 24 hour time points and very slightly higher at three hours (Table 3). The combined blood cell quantitation value differences between incubation conditions, shaking and stationary, at 56°C and 65°C were also small and did not show significance when time was not considered (S5 Table). The increase in yields after one hour of incubation between the temperatures over time up to 18 hours with and without shaking, while observed, were not large (S5 Table). All of the blood samples, whether incubated stationary or shaken and at 56°C or 65°C, showed decreased average DNA yields at 24 hours (Table 3). These drops in yield, combined across temperature and shaking conditions reached significance (F_(1,3)_ = 934.21, *p*-value = 0.001) (S5 Table). The highest average quantitation values for blood cells that were not re-suspended were 1.45±0.33 ng/µL for the samples incubated for three hours at 56°C with shaking and 1.47±0.02 ng/µL for the samples incubated for three hours at 65°C with shaking (Table 3).

The combined average quantities of DNA recovered from buccal and blood cells at 24 hours of incubation across all conditions were significantly lowered when compared to the combined values from the one, three, and 18 hour experiments (F_(3,105)_ = 10.18, *p*-value<0.001), see Tables 2 and 3, and S6 Table.

### Swab Re-suspension

Buccal and blood swab samples were sequentially re-suspended in their extraction buffer by centrifugation with DNA IQ Spin Baskets to find out if the repeated “washing” of the swab with the buffer would yield more usable DNA. The samples were first tested using the standard protocol parameters of a one hour incubation at 56°C with shaking at 900 rpm. Further tests were done, individually and in combination, without shaking, at 65°C, and for three hours of incubation. Time points beyond three hours were not practical with the re-suspension method due to the number of sample manipulations that would have been required. The results are shown in Table 2 (buccal cells) and Table 3 (blood cells). Every variation of the incubation conditions demonstrated higher average yields of DNA with swab re-suspension than the same set of conditions without re-suspension. In some instances the increase was small, such as buccal cells incubated stationary at 56°C for one hour (0.26±0.02 ng/µL) versus the same conditions with re-suspension (0.37±0.08 ng/µL). However, there were some sample sets that showed substantial gains, such as blood cells incubated with shaking at 56°C for one hour (0.64±0.33 ng/µL) versus the same conditions with re-suspension (1.87±0.42 ng/µL) (Table 3), which approached significance (S7 Table). Buccal cells also had increased yield with re-suspension, with an average recovered yield when incubated with shaking at 56°C for one hour with re-suspension of 1.32±0.51 ng/µL versus the same incubation conditions without re-suspension of 0.54±0.06 ng/µL (Table 2). The increased yield for buccal cells did not reach significance, however (S7 Table). The average quantitation results for buccal and blood cell samples incubated with shaking at 56°C for one hour and extracted in liquid form, from swabs, and from swabs with re-suspension are shown for comparison in Fig. 1.

### Degradation and Contamination

All of the extracted samples were amplified and their genotypes were assessed for accuracy and contamination. All of the samples showed the expected genotype and no contamination was observed. S1 Fig. is a representative electropherogram of a buccal cell sample dried onto a swab and extracted immediately using the standard protocol conditions (shaking at 56°C for one hour) and demonstrates the full genotype. Evidence of DNA degradation was seen in samples that were incubated for 24 hours. Decreased peak heights in larger loci were observed for buccal and blood cell samples (see S2 Fig. for an example). In addition to decreased peak heights, allele drop-out was observed (allele 22 in locus D18S51) in one of the blood cell samples incubated stationary at 65°C for 24 hours (see S2 Fig.). The possibility of DNA degradation was also examined by calculating the peak balance for sister alleles in heterozygous loci and the peak heights across the loci for all of the extraction conditions. No evidence of DNA degradation was found for samples incubated up to 18 hours at either temperature with or without shaking, all heterozygous peak pairs had a balance of ≥70%, but imbalance was present in some of the larger loci in buccal and blood cell samples that were incubated for 24 hours (see S2 Fig. as an example). The swab re-suspension method did not induce DNA degradation. S3 Fig. is a representative electropherogram of a blood cell sample incubated with shaking (900 rpm) at 65°C for three hours with swab re-suspension. Heterozygous peak height balance values were consistent with those seen at the equivalent extraction conditions without swab re-suspension (≥70%, see S3 Fig. as an example).

### Storage

All of the swab samples used in this study had been made, dried overnight, and then extracted within 72 hours. In order to assess if storage for longer periods of time would have any substantial influence on the quantity or quality of recovered DNA using the standard protocol (shaking at 56°C for one hour) or the standard protocol plus swab re-suspension, buccal and blood swabs were created and stored as previously described. Average recovered quantities from aged samples did not significantly differ from those seen with the same relative extraction method performed on the freshly prepared swabs. The average recovered quantitation value from buccal cells aged for six months on a swab and extracted with re-suspension was 1.17±0.22 ng/µL and, compared to an average value for fresh samples extracted with re-suspension of 1.32±0.51 ng/µL, was not significantly different (S8 Table). The average recovered quantitation value from blood cells aged for six months on a swab and extracted with re-suspension was 1.98±0.29 ng/µL, compared to an average value for fresh samples extracted with re-suspension of 1.87±0.42 ng/µL (S8 Table). The quantitation data for the samples aged for one week, one month, and three months and extracted with re-suspension and for all of the time point samples extracted with the standard protocol demonstrated results similar to those for six months. The aged samples did not demonstrate signs of DNA degradation, either. S4 Fig. is a representative electropherogram of a buccal cell sample dried onto a swab and stored for six months 4°C and extracted at 56°C for one hour with shaking at 900 rpm with re-suspension.

## Discussion

Comparison of the quantitation values for DNA recovered from the liquid buccal and blood cell samples to those from an equal volume dried onto cotton swabs indicates that a large portion of the DNA is lost. Some of the DNA may not bind to the silica gel in the extraction columns for collection, or may be lost to non-specific binding to the polypropylene collection tubes, however this work demonstrates that much of it is retained on the swab. This condition is not necessarily challenging for forensic DNA casework on high quality and quantity single source samples, where more than enough DNA has been recovered to perform multiple analyses. Many casework samples, however, are low quantity, mixtures, degraded, carry PCR inhibitors, or are combinations of these factors. When working with these types of challenged samples, recovering as much DNA as possible becomes critical to mitigating, or avoiding altogether, the effects of stochastic amplification that can adversely affect data interpretation. The data from our experiments has demonstrated that altering the extraction conditions can lead to increased yields from samples collected on cotton swabs, but can also decrease them. Therefore, this study has demonstrated that at least small increases can be achieved through modifications of the lysis step, however these modifications should be evaluated by individual laboratories since the results were highly variable and some led to a loss in DNA yield.

The DNA extraction kit manufacturer recommends that cotton swabs be incubated at 56°C with shaking at 900 rpm “for at least 1 hour” [28]. Results indicated that shaking the samples during incubation will usually yield increased quantities of DNA, although the differences are not always large. The blood cell samples incubated for three hours at 56°C had disparate average quantitation values based on agitation: 1.45±0.33 ng/µL when shaken versus 0.56±0.17 ng/µL when stationary. The buccal cell samples incubated for three hours at 56°C had nearly identical average quantitation values, regardless of shaking: 0.31 ng/µL±0.07 versus 0.30±0.02 ng/µL. This range of results indicates that the buffer agitation that comes from shaking may be important for some samples, perhaps because the DNA is more deeply embedded in the cotton fibers, and less so for others. Variations in temperature were also shown to have less impact on recovered DNA yield from samples that were shaken. Buccal cell samples had their highest average yield when extracted for 18 hours at 65°C with shaking (0.65±0.01 ng/µL), however under the same conditions at 56°C the yield was not greatly different (0.44±0.11 ng/µL) (Table 2). Blood cell samples were even closer, with nearly indistinguishable maximum average yields of 1.45±0.33 ng/µL at 56°C and 1.47±0.02 ng/µL at 65°C, both after three hours of incubation (Table 3). The incubation time proved to be more important to DNA yield than shaking or temperature variations. All of the incubation conditions except for buccal cells shaken at 56°C demonstrated increased average DNA yields by increasing the incubation time past one hour to three or 18 hours (Tables 2 and 3). However, there were significant (F_(3,105)_ = 10.18, *p*-value<0.001) (S6 Table) reductions in yields for all of the sample extraction conditions at 24 hours when compared to the combined average yields recovered at one, three, and 18 hours for all temperature and shaking conditions. Not only was less DNA recovered after 24 hours of incubation, the quality was also reduced, with lower allelic peak heights in some samples. Large locus allelic dropout was also observed in a blood cell sample incubated stationary at 65°C. This result was expected due to the low quantity of DNA recovered (<100 pg/µL) for these samples, compared to the quantities recovered at the shorter incubation times. All of the buccal or blood samples started with approximately equal amounts of DNA in their respective groups, so the decreased recovery could be due to retention on the swab and/or DNA degradation. Both likely contribute, with degradation to the point of large locus allelic dropout occurring in samples extracted with the combination of 24 hours incubation at an increased temperature of 65°C.

The swab re-suspension method improved the average yield of DNA recovered from cotton swabs under every condition examined for each of the cell types (Tables 2 and 3). Comparing average yield values from extraction with the standard protocol conditions (0.54±0.06 ng/µL) to those from the standard protocol plus swab re-suspension (1.32±0.51 ng/µL), buccal cell samples had an increase of approximately two-fold in recovered DNA (Table 2 and Fig. 1). The average yields with blood cells increased more, with an approximately three-fold gain from the standard protocol conditions (0.64±0.33 ng/µL) to those from the standard protocol plus swab re-suspension (1.87±0.42 ng/µL) (Table 3 and Fig. 1). The results were more variable with buccal cells, possibly due to the less homogenous nature of the cell suspension as opposed to the blood samples. Increasing the incubation temperature in combination with swab re-suspension did not usually increase the yields, for most samples there were slight decreases in average quantities of recovered DNA for both one and three hour incubation times (Tables 2 and 3). The exceptions were the buccal and blood cell samples incubated for three hours with shaking, which both showed slight increases in yield at 65°C (Tables 2 and 3, and S9 Table). Increasing the incubation time in combination with swab re-suspension did increase the average recovered yield for every protocol used with the exception of the buccal cells shaken at 56°C, which decreased from an average of 1.32±0.51 ng/µL at one hour to an average of 0.71±0.25 ng/µL at three hours. The increase in recovered DNA yield detected at three hour incubations was not unexpected, as the swabs were re-suspended every 20 minutes, so the swabs incubated for three hours had more buffer washes than those at one hour, and therefore more opportunity to dislodge DNA molecules retained on the swab.

The sample manipulations required by the swab re-suspension process did not cause detectable DNA degradation, nor did the samples show any signs of contamination (S3 Fig.). The process does not include any vortexing steps, or other ways to induce excessive shearing stress to the DNA molecules. It is similar, in principle, to the differential extraction procedure first described by Gill et al. [30] and modified by forensic laboratories for casework samples that contain sperm cells. The differential procedure uses repeated washes to remove epithelial and sperm cells from evidentiary swabs, and then additional washes to remove non-sperm DNA from the pelleted sperm cells. Those washes are often discarded, along with any DNA that may be in them. The swab re-suspension method presented in this work sequentially uses the extraction buffer as the wash to dislodge cells that are caught in, or adhered to, the cotton fibers of the swab. As no buffer is discarded, DNA is not lost. The swab re-suspension method also proved to be effective with samples that had been stored up to six months at 4°C. Under these conditions the DNA should not have been significantly degraded and testing supported that the swab re-suspension method did not cause additional DNA damage to aged samples (compare S1 and S4 Figs.) while increasing yield.

## Conclusions

This research was intended to test alterations to a standard DNA extraction protocol to discover if any simple changes could increase the yield of DNA recovered from cotton tipped swabs used to collect evidentiary samples. We have shown that the most critical component of the extraction protocol to alter is the time of incubation. The additional incubation times were chosen in consideration of the practical demands of an eight hour work day and the potential practice of incubating samples overnight. An incubation time increase to at least three hours generally yielded equal or higher quantities of typable DNA and extraction incubations up to 18 hours were not deleterious to DNA recovery. As a result, increasing the extraction incubation time to three hours is recommended. Incubations up to 18 hours can be done without compromising yield, however further gains in DNA recovery are usually minimal, if existent at all. At incubations of 24 hours, however, DNA yield dropped substantially for most samples. DNA degradation was also observed in a sample incubated for 24 hours combined with an increase in incubation temperature to 65°C. Because of these results, DNA extraction incubations of 24 hours or more are not recommended.

An additional step in the extraction process was also developed that led to increased recovery of DNA from cotton swabs. By sequentially washing and separating the extraction buffer from the swab, we were able to increase DNA yields by more than two-fold for some samples. When processing low quantity and/or quality forensic DNA samples, the difference of only a few picograms can determine if a full or partial profile is developed. For that reason, altering the extraction protocol to increase DNA yields by even small margins could be highly beneficial. The alterations and additions we have described can also be used with a variety of DNA extraction methods, kits, and types of swabs to further increase recovered DNA yields.

## Supporting Information

S1 Fig **Electropherogram of a buccal cell sample dried onto a swab and extracted immediately using the standard protocol conditions of incubation for 1 hour at 56°C with shaking at 900 rpm without re-suspension.**
(TIF)

S2 Fig **Electropherogram of a blood cell sample demonstrating DNA degradation.** The swab was incubated stationary at 65°C for 24 hours and extracted without re-suspension. Peak heights decrease as locus size increases and allele 22 has dropped out at locus D18S51 (position indicated by arrow).
(TIF)

S3 Fig **Electropherogram of a blood cell sample extracted with the swab re-suspension method.** The swab was incubated for 3 hours at 65°C with shaking at 900 rpm. Peak heights and peak balance within and between loci show no indications of DNA degradation using this method.
(TIF)

S4 Fig **Electropherogram of a buccal cell sample stored for 6 months at 4°C and extracted with the swab re-suspension method.** The swab was incubated for 1 hour at 56°C with shaking at 900 rpm. Peak heights and peak balance within and between loci show no indications of DNA degradation after storage.
(TIF)

S1 Table ***p*** **-values for average recovered DNA quantities from liquid buccal and blood cell samples compared to equal volumes dried onto cotton swabs.** All samples were incubated using the recommended extraction protocol (1 hour, 56°C, shaken) without swab re-suspension.
(DOCX)

S2 Table ***p*** **-values for average recovered DNA quantities from swabs with buccal cell samples incubated at 56°C with alterations to the extraction protocol as described without re-suspension.**
(DOCX)

S3 Table ***p*** **-values for average recovered DNA quantities from swabs with blood cell samples incubated at 56°C with alterations to the extraction protocol as described without re-suspension.**
(DOCX)

S4 Table ***p*** **-values for average recovered DNA quantities from swabs with buccal cell samples incubated at 65°C with alterations to the extraction protocol as described without re-suspension.**
(DOCX)

S5 Table ***p*** **-values for average recovered DNA quantities from swabs with blood cell samples incubated at 65°C with alterations to the extraction protocol as described without re-suspension.**
(DOCX)

S6 Table ***p*** **-value for average recovered combined DNA quantities from swabs with buccal or blood cell samples incubated across all conditions over time without re-suspension.**
(DOCX)

S7 Table ***p*** **-values for average recovered DNA quantities from swabs with buccal or blood cell samples incubated using the recommended extraction protocol (1 hour, 56°C, shaken) with and without the swab re-suspension extraction protocol.**
(DOCX)

S8 Table ***p*** **-values for average recovered DNA quantities from buccal and blood cell samples stored on swabs for six months and incubated using the recommended extraction protocol (1 hour, 56°C, shaken) with re-suspension.**
(DOCX)

S9 Table ***p*** **-values for average recovered DNA quantities from swabs with buccal or blood cell samples incubated at 56°C with alterations to the extraction protocol as described with re-suspension.**
(DOCX)
