# Supplementary figures and images for: Evaluation of Methods to Improve the Extraction and Recovery of DNA from Cotton Swabs for Forensic Analysis
Source: PLoS One. 2014 Dec 30;9(12):e116351. doi: 10.1371/journal.pone.0116351 (PMC4280208; doi:10.1371/journal.pone.0116351)

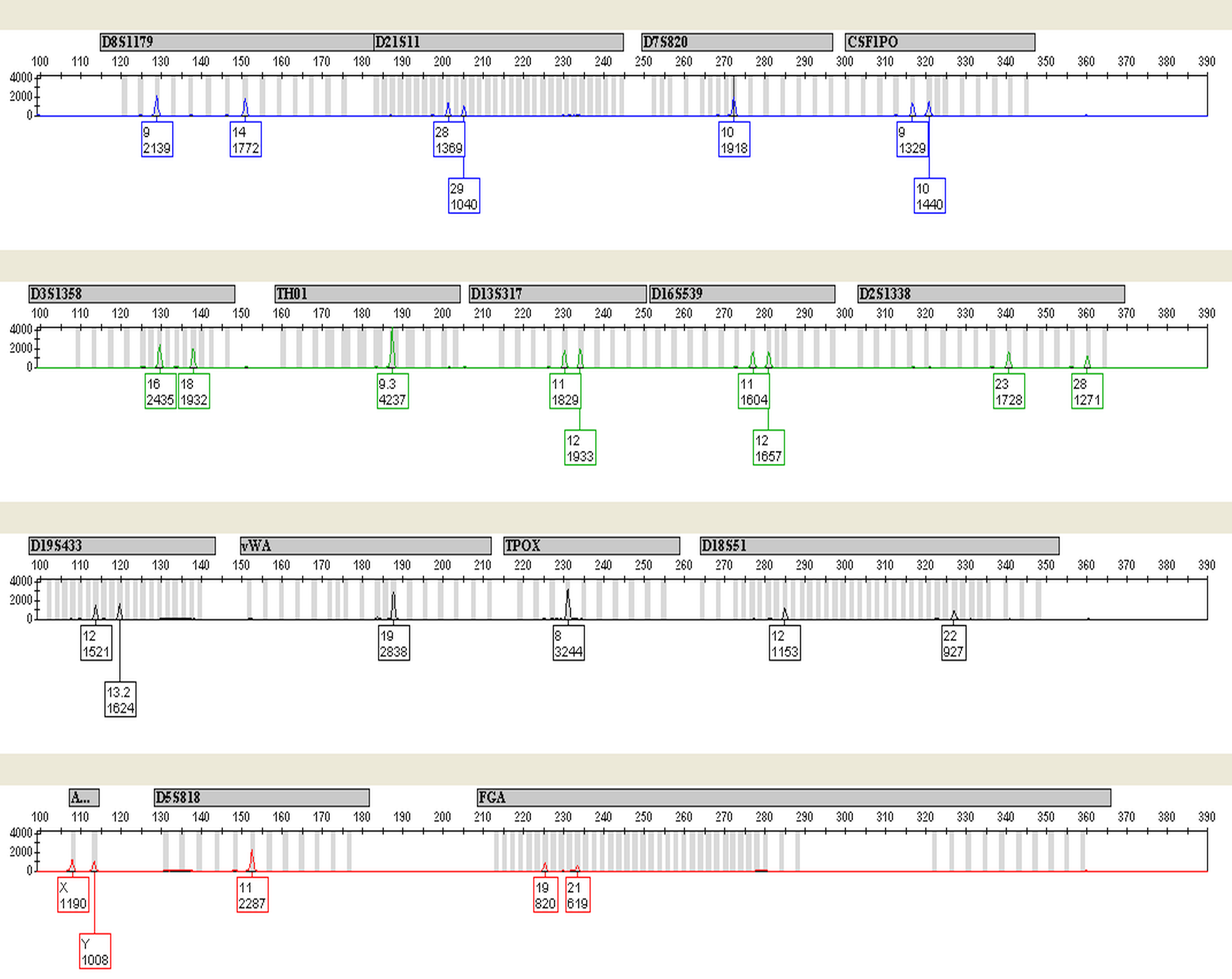

Supplement: S1 Fig — Electropherogram of a buccal cell sample dried onto a swab and extracted immediately using the standard protocol conditions of incubation for 1 hour at 56°C with shaking at 900 rpm without re-suspension. (TIF) [file pone.0116351.s001.tif]

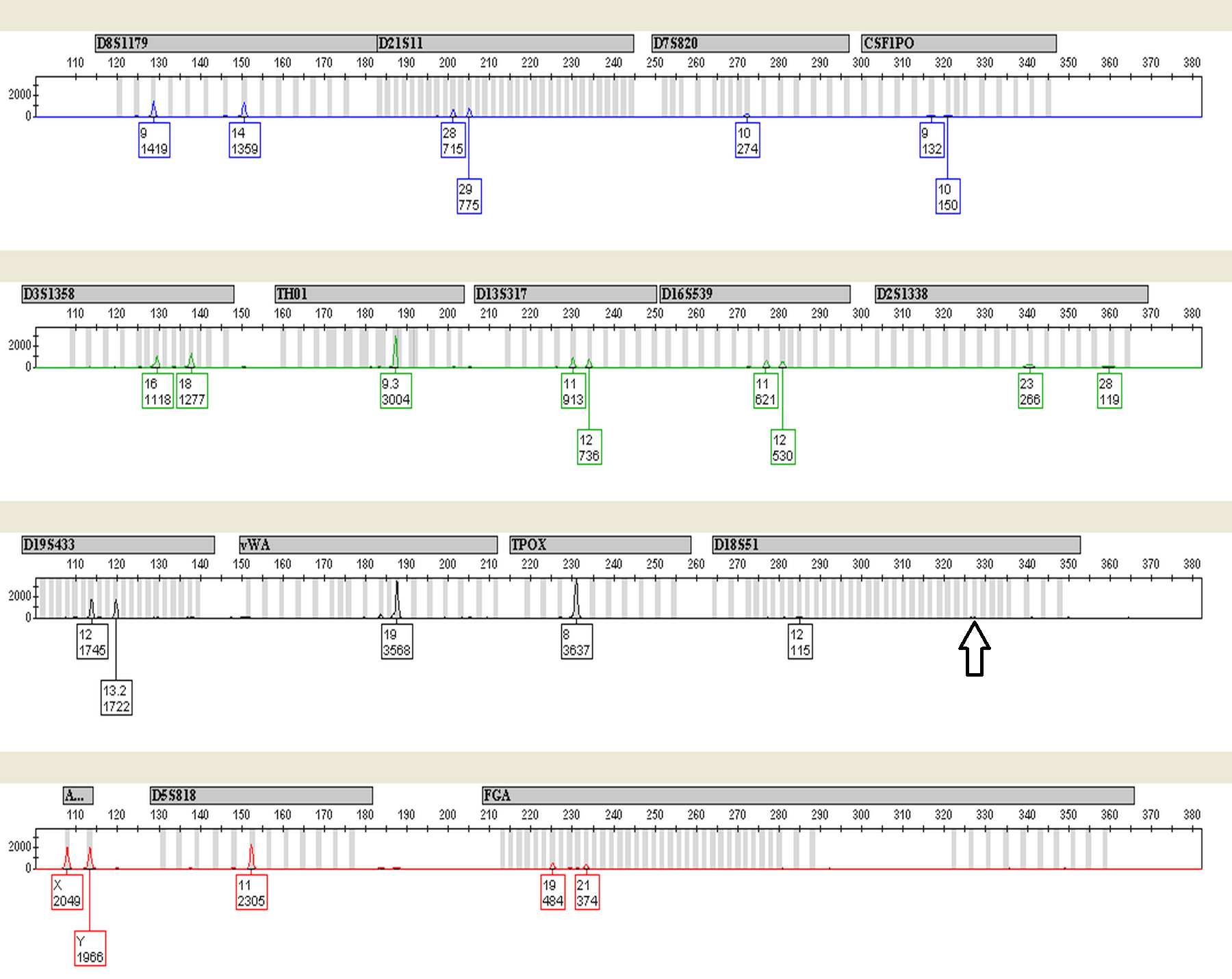

Supplement: S2 Fig — Electropherogram of a blood cell sample demonstrating DNA degradation. The swab was incubated stationary at 65°C for 24 hours and extracted without re-suspension. Peak heights decrease as locus size increases and allele 22 has dropped out at locus D18S51 (position indicated by arrow). (TIF) [file pone.0116351.s002.tif]

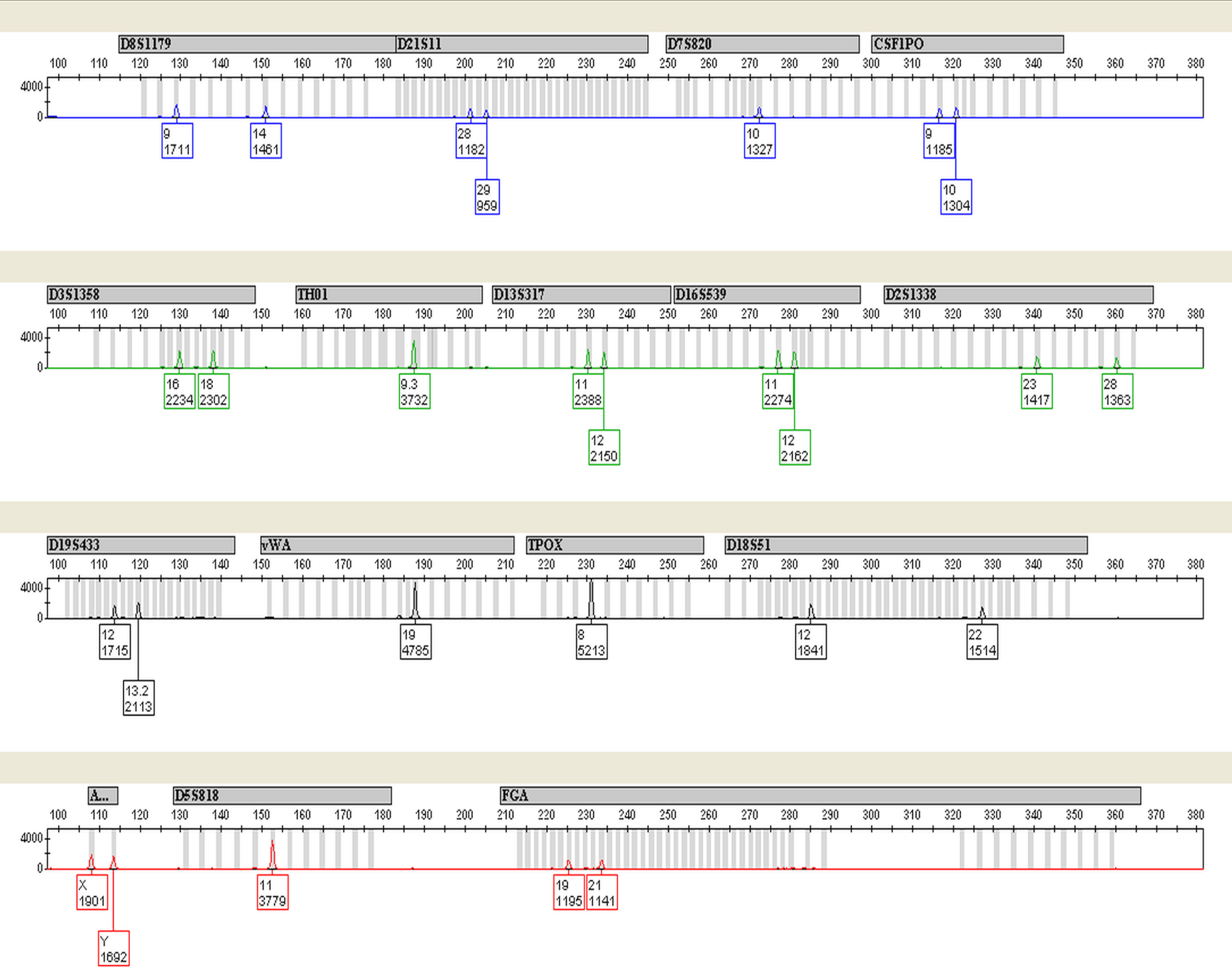

Supplement: S3 Fig — Electropherogram of a blood cell sample extracted with the swab re-suspension method. The swab was incubated for 3 hours at 65°C with shaking at 900 rpm. Peak heights and peak balance within and between loci show no indications of DNA degradation using this method. (TIF) [file pone.0116351.s003.tif]

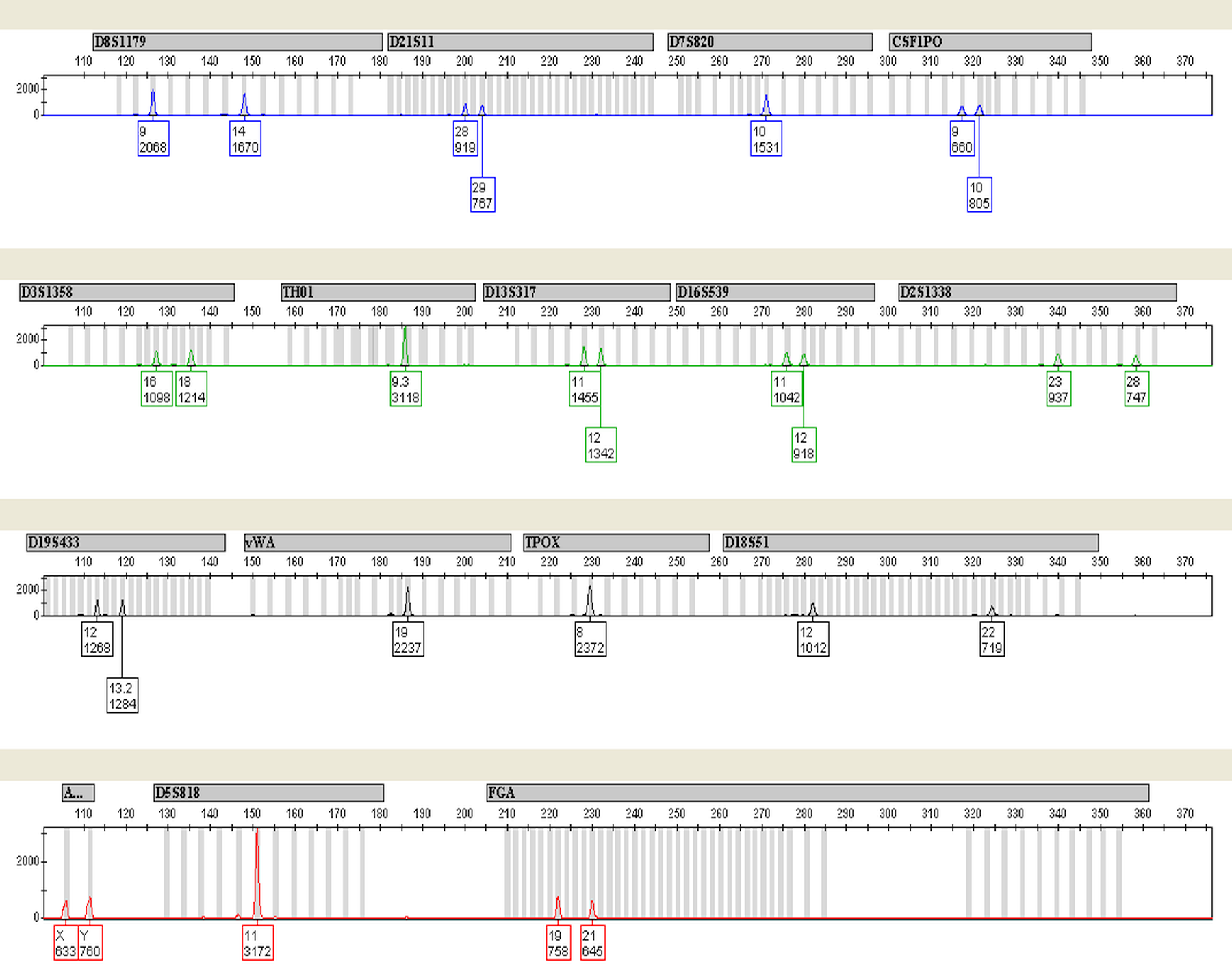

Supplement: S4 Fig — Electropherogram of a buccal cell sample stored for 6 months at 4°C and extracted with the swab re-suspension method. The swab was incubated for 1 hour at 56°C with shaking at 900 rpm. Peak heights and peak balance within and between loci show no indications of DNA degradation after storage. (TIF) [file pone.0116351.s004.tif]
