# Supplementary material for: Evaluation of Methods to Improve the Extraction and Recovery of DNA from Cotton Swabs for Forensic Analysis
Source: PLoS One. 2014 Dec 30;9(12):e116351. doi: 10.1371/journal.pone.0116351 (PMC4280208; doi:10.1371/journal.pone.0116351)
Supplement: S1 Table — p -values for average recovered DNA quantities from liquid buccal and blood cell samples compared to equal volumes dried onto cotton swabs. All samples were incubated using the recommended extraction protocol (1 hour, 56°C, shaken) without swab re-suspension. (DOCX) [file pone.0116351.s005.docx]

Table S1. *p*-values for average recovered DNA quantities from liquid buccal and blood cell samples compared to equal volumes dried onto cotton swabs. All samples were incubated using the standard extraction protocol (1 hour, 56˚C, shaken) without swab re-suspension.

| Condition | Compared Condition | *p*-value | Significant |
| --- | --- | --- | --- |
| Liquid buccal cell suspension | Buccal cell suspension dried on swab | 0.002 | Yes |
| Liquid blood | Blood dried on swab | <0.001 | Yes |
