# Supplementary material for: Evaluation of Methods to Improve the Extraction and Recovery of DNA from Cotton Swabs for Forensic Analysis
Source: PLoS One. 2014 Dec 30;9(12):e116351. doi: 10.1371/journal.pone.0116351 (PMC4280208; doi:10.1371/journal.pone.0116351)
Supplement: S3 Table — p -values for average recovered DNA quantities from swabs with blood cell samples incubated at 56°C with alterations to the extraction protocol as described without re-suspension. (DOCX) [file pone.0116351.s007.docx]

Table S3. *p*-values for average recovered DNA quantities from swabs with blood cell samples incubated at 56˚C with alterations to the extraction protocol as described without re-suspension.

| Condition | Compared Condition | *p*-value | Significant |
| --- | --- | --- | --- |
| 1 hour, shaken | 1 hour, stationary | 0.761 | No |
| 3 hours, shaken | 3 hours, stationary | 0.398 | No |
| 18 hours, shaken | 18 hours, stationary | 0.128 | No |
| 1 hour, shaken | Combined 3 and 18 hours, shaken | 0.305 | No |
| 3 hours, shaken | 24 hours, shaken | 0.314 | No |
| 1 hour, stationary | Combined 3, 18, and 24 hours, stationary | 0.202 | No |
