# Supplementary material for: Evaluation of Methods to Improve the Extraction and Recovery of DNA from Cotton Swabs for Forensic Analysis
Source: PLoS One. 2014 Dec 30;9(12):e116351. doi: 10.1371/journal.pone.0116351 (PMC4280208; doi:10.1371/journal.pone.0116351)
Supplement: S4 Table — p -values for average recovered DNA quantities from swabs with buccal cell samples incubated at 65°C with alterations to the extraction protocol as described without re-suspension. (DOCX) [file pone.0116351.s008.docx]

Table S4. *p*-values for average recovered DNA quantities from swabs with buccal cell samples incubated at 65˚C with alterations to the extraction protocol as described without re-suspension.

| Condition | Compared Condition | *p*-value | Significant |
| --- | --- | --- | --- |
| Combined shaken and stationary | 56˚C, combined shaken and stationary | 0.808 | No |
| 1 hour, combined 65˚C, 56˚C, shaken, and stationary | Combined 3 hours, 18 hours, 65˚C, 56˚C, shaken, stationary | 0.001 | Yes |
