# Supplementary material for: Evaluation of Methods to Improve the Extraction and Recovery of DNA from Cotton Swabs for Forensic Analysis
Source: PLoS One. 2014 Dec 30;9(12):e116351. doi: 10.1371/journal.pone.0116351 (PMC4280208; doi:10.1371/journal.pone.0116351)
Supplement: S6 Table — p -value for average recovered combined DNA quantities from swabs with buccal or blood cell samples incubated across all conditions over time without re-suspension. (DOCX) [file pone.0116351.s010.docx]

Table S6. *p*-values for average recovered combined DNA quantities from swabs with buccal or blood cell samples incubated across all conditions over time without re-suspension.

| Condition | Compared Condition | *p*-value | Significant |
| --- | --- | --- | --- |
| Combined 1 hour, 3 hour, 18 hour, 65˚C, 56˚C, shaken and stationary | 24 hours, combined 65˚C, 56˚C, shaken and stationary | <0.001 | Yes |
