# Supplementary material for: Evaluation of Methods to Improve the Extraction and Recovery of DNA from Cotton Swabs for Forensic Analysis
Source: PLoS One. 2014 Dec 30;9(12):e116351. doi: 10.1371/journal.pone.0116351 (PMC4280208; doi:10.1371/journal.pone.0116351)
Supplement: S7 Table — p -values for average recovered DNA quantities from swabs with buccal or blood cell samples incubated using the recommended extraction protocol (1 hour, 56°C, shaken) with and without the swab re-suspension extraction protocol. (DOCX) [file pone.0116351.s011.docx]

Table S7. *p*-values for average recovered DNA quantities from swabs with buccal or blood cell samples incubated using the recommended extraction protocol (1 hour, 56˚C, shaken) with and without the swab re-suspension extraction protocol.

| Condition | Compared Condition | *p*-value | Significant |
| --- | --- | --- | --- |
| Blood cells, no re-suspension | Blood cells, with re-suspension | 0.054 | No |
| Buccal cells, no re-suspension | Buccal cells, with re-suspension | 0.414 | No |
