# Supplementary material for: Evaluation of Methods to Improve the Extraction and Recovery of DNA from Cotton Swabs for Forensic Analysis
Source: PLoS One. 2014 Dec 30;9(12):e116351. doi: 10.1371/journal.pone.0116351 (PMC4280208; doi:10.1371/journal.pone.0116351)
Supplement: S8 Table — p -values for average recovered DNA quantities from buccal and blood cell samples stored on swabs for six months and incubated using the recommended extraction protocol (1 hour, 56°C, shaken) with re-suspension. (DOCX) [file pone.0116351.s012.docx]

Table S8. *p*-values for average recovered DNA quantities from buccal and blood cell samples stored on swabs for six months and incubated using the recommended extraction protocol (1 hour, 56˚C, shaken) with re-suspension.

| Condition | Compared Condition | *p*-value | Significant |
| --- | --- | --- | --- |
| Stored buccal cells | Fresh buccal cells | 0.884 | No |
| Stored blood cells | Fresh blood cells | 0.803 | No |
