# Supplementary material for: Evaluation of Methods to Improve the Extraction and Recovery of DNA from Cotton Swabs for Forensic Analysis
Source: PLoS One. 2014 Dec 30;9(12):e116351. doi: 10.1371/journal.pone.0116351 (PMC4280208; doi:10.1371/journal.pone.0116351)
Supplement: S9 Table — p -values for average recovered DNA quantities from swabs with buccal or blood cell samples incubated at 56°C with alterations to the extraction protocol as described with re-suspension. (DOCX) [file pone.0116351.s013.docx]

Table S9. *p*-values for recovered average DNA quantities from swabs with buccal or blood cell samples incubated at 56˚C with alterations to the extraction protocol as described with re-suspension.

| Condition | Compared Condition | *p*-value | Significant |
| --- | --- | --- | --- |
| Buccal cells, 3 hours, 56˚C, shaken | Buccal cells, 3 hours, 65˚C, shaken | 0.709 | No |
| Blood cells, 3 hours, 56˚C, shaken | Blood cells, 3 hours, 65˚C, shaken | 0.907 | No |
